# Bilirubin metabolism-related genetic variants in neonatal hyperbilirubinemia: a multicenter case-control study in northern China

**DOI:** 10.3389/fped.2026.1945311

**Published:** 2026-10-20

**Authors:** Sihan Sheng, Ying Li, Lijuan Ma, Hesheng Chang, Yanmei Guan, Yanzhe Lei, Chunying Tan, Arigonggaowa, Hongfei Wen, Chen Kou, Yajuan Wang

**Affiliations:** 1 Capital Institute of Pediatrics, Chinese Academy of Medical Sciences & Peking Union Medical College, Beijing, China; 2 Department of Neonatology, Capital Center for Children’s Health, Capital Medical University, Capital Institute of Pediatrics, Beijing, China; 3 Laboratory Center, Capital Center for Children’s Health, Capital Medical University, Capital Institute of Pediatrics, Beijing, China; 4 Department of Pediatrics, Beijing Chaoyang Hospital, Capital Medical University, Beijing, China; 5 Department of Obstetrics, The First Hospital of Tsinghua University, School of Clinical Medicine Tsinghua Medicine Tsinghua University, Beijing, China; 6 Neonatal Department, Haidian District Maternal and Child Health Care Hospital, Beijing, China; 7 Neonatal Department, Liaoning Maternal and Child Health Hospital, Shenyang, Liaoning, China; 8 Neonatal Department, Inner Mongolia Maternity and Child Health Care Hospital Neonatology Division, Hohhot, Inner Mongolia, China; 9 Department of Pediatrics, Beijing Aerospace General Hospital, Beijing, China; 10 Neonatal Department, Beijing Obstetrics and Gynecology Hospital, Capital Medical University, Beijing, China

**Keywords:** bilirubin metabolism, genetic variant, neonatal hyperbilirubinemia, northern China, *UGT1A1*

## Abstract

Neonatal hyperbilirubinemia (NHB) is common in neonates, and genetic factors are important contributors to bilirubin metabolism. However, data on NHB-related genetic variants in northern China remain limited. This multicenter case-control study included 413 neonates hospitalized with unexplained NHB and 120 control neonates who had not been diagnosed with hyperbilirubinemia at enrollment on the day of birth. Twelve hotspot loci in *UGT1A1*, *G6PD*, *SLC25A13*, *SLC10A1*, and *ABCC2* were genotyped using multicolor melting curve analysis. The overall target variant detection rate was significantly higher in cases than in controls (63.0% vs. 45.0%; *OR* = 2.08, 95% *CI*: 1.38–3.13, *P* < 0.001). *UGT1A1* variants were the most frequently detected and were more common in cases than in controls (*OR* = 2.20, 95% *CI*: 1.45–3.33, *P* < 0.001). *UGT1A1* c.211G>A was the predominant locus, and carriers of at least one variant allele were more frequent in cases than in controls (53.3% vs. 37.5%; *OR* = 1.90, 95% *CI*: 1.25–2.89, *P* = 0.002), with the association remaining significant after FDR correction. Multi-site variants were detected in 34 cases and 5 controls. Among these neonates, 25 had within-gene multi-site variants, all involving *UGT1A1*, whereas 14 had cross-gene multi-site variants, mainly involving coexistence of *UGT1A1* variants with *SLC10A1* or *G6PD* variants. These findings provide regional data on the distribution of bilirubin metabolism-related genetic variants in northern Chinese neonates with unexplained NHB.

## 1 Introduction

Neonatal hyperbilirubinemia (NHB) is a common clinical condition in the neonatal period. Approximately 60% of term neonates and 80% of preterm neonates develop jaundice of varying degrees. Elevated bilirubin levels, especially excessive unconjugated bilirubin, can be neurotoxic. Severe cases may cause hearing and visual impairment and may even lead to serious long-term neurological sequelae (1–3). The etiology of NHB is complex. Common contributing factors include hemolytic disease due to maternal-fetal blood group incompatibility, red blood cell enzyme defects, erythrocyte morphological abnormalities, extravascular hemolysis, infection, and delayed meconium passage. Nevertheless, a considerable proportion of affected neonates have no clearly identifiable cause.

With advances in genomic research, genetic variants have been increasingly recognized as contributors to NHB by affecting bilirubin production, hepatocellular uptake, glucuronidation, and biliary excretion (4). Among the genes involved in bilirubin metabolism, *UGT1A1* is one of the most extensively studied and clinically relevant genes. The UGT1A1 enzyme encoded by this gene is the key rate-limiting enzyme that catalyzes the conjugation of unconjugated bilirubin with glucuronic acid to form water-soluble conjugated bilirubin. *UGT1A1* variants can reduce or abolish enzyme activity, thereby increasing the risk of hyperbilirubinemia (5, 6).

In addition to *UGT1A1*, several other genes may also contribute to genetic susceptibility to NHB. Defects in the X-linked *G6PD* gene are important genetic causes of neonatal hemolytic jaundice, mainly by inducing hemolysis and increasing bilirubin production (7, 8). *SLC10A1* encodes sodium taurocholate cotransporting polypeptide (NTCP), which participates in hepatocellular uptake of bile acids and bilirubin-related metabolites. *SLC25A13* encodes the citrin protein, and its deficiency is associated with neonatal intrahepatic cholestasis caused by citrin deficiency (NICCD). *ABCC2* encodes multidrug resistance-associated protein 2, which is involved in biliary excretion of conjugated bilirubin; variants in this gene can cause Dubin-Johnson syndrome (9, 10). Together, these genes form a complex genetic regulatory network related to bilirubin metabolism and transport.

Notably, variants in bilirubin metabolism-related genes exhibit marked ethnic and geographic differences (11). In Caucasian populations, the *UGT1A1* promoter TA repeat insertion variant UGT1A1*28 [A(TA)7TAA] is most common, whereas in East Asian populations, the coding-region variant *UGT1A1* c.211G>A (p.Gly71Arg) is more frequent (12). Similarly, common *G6PD* variants differ substantially across regions and ethnic groups. Newborn screening studies in China have shown that the prevalence of G6PD deficiency is higher in southern than in northern China; however, G6PD deficiency and related gene variants can still be detected in northern populations (8, 13–15). Therefore, characterizing region-specific distributions of bilirubin metabolism-related variants is important for understanding the genetic background of NHB.

At present, data on the genetic variant spectrum associated with NHB in northern China remain limited. This multicenter case-control study aimed to characterize the regional distribution of 12 hotspot loci in five bilirubin metabolism-related genes (*UGT1A1*, *G6PD*, *SLC25A13*, *SLC10A1*, and *ABCC2*) and explore their associations with unexplained NHB in northern Chinese neonates.

## 2 Materials and methods

### 2.1 Study population

This multicenter case-control study was conducted across seven hospitals in northern China between October 2023 and May 2025. All enrolled neonates had a gestational age of ≥35 weeks and were included during the neonatal period (within 28 days after birth). Cases were recruited from all seven participating hospitals, whereas controls were recruited from six hospitals during the same study period. The distribution of participants across centers is summarized in Supplementary Table S1.

The case group comprised 413 neonates hospitalized with unexplained NHB. NHB was defined as a total serum bilirubin (TSB) level above the 95th percentile for postnatal age on the hour-specific bilirubin nomogram. Cases were characterized predominantly by unconjugated bilirubin elevation, with no common clinical cause identified after routine etiological evaluation. Neonates with hemolytic disease, infection, polycythemia, clinically significant inadequate intake, or delayed meconium passage were excluded (16–18). Additional exclusion criteria included previous allogeneic blood transfusion, congenital malformations, a known family history of hereditary disease, and clinically suspected or confirmed genetic disorders; target-variant carriage alone was not an exclusion criterion.

The control group included 120 neonates selected from eligible neonates born in the obstetric departments during the same period who had not been diagnosed with NHB at enrollment on the day of birth. Controls had normal Apgar scores and no history of asphyxia and were subject to the same general exclusion criteria regarding transfusion, congenital malformations, a known family history of hereditary disease, and suspected or confirmed genetic disorders. Because controls were enrolled at birth, they were defined by the absence of diagnosed hyperbilirubinemia at enrollment rather than throughout the neonatal period.

Exploratory telephone follow-up was performed only for control neonates with detected target variants and was used descriptively without reclassification of control status. Written informed consent was obtained from the legal guardians of all participants. The study protocol was approved by the Ethics Committee of Capital Institute of Pediatrics (approval no. SHERLL2024034-01, Beijing, China) and was conducted in accordance with the Declaration of Helsinki.

### 2.2 Study methods

#### 2.2.1 Selection of genes and loci

Based on previous genetic evidence, reported relevance to bilirubin metabolism or NHB, and population-specific considerations, we selected 12 hotspot variants in five bilirubin metabolism-related genes (*UGT1A1*, *G6PD*, *SLC25A13*, *SLC10A1*, and *ABCC2*). These genes were selected to represent different aspects of bilirubin metabolism and transport, despite the predominance of unconjugated hyperbilirubinemia in the study cohort. Variant nomenclature was verified against the reference transcripts used for assay annotation and the GRCh38 reference genome. Reference transcript accession numbers and dbSNP identifiers are provided in Table 1.

**Table 1:** Annotation and allele frequencies of the 12 targeted variants.

Gene	Variant locus	Reference transcript	dbSNP ID	Case group allele frequency (%)	Control group allele frequency (%)
*UGT1A1*	c.211G>A (p.Gly71Arg)	NM_000463.3	rs4148323	32.93	21.25
*UGT1A1*	c.686C>A (p.Pro229Gln)	NM_000463.3	rs35350960	0.85	0.42
*UGT1A1*	c.1091C>T (p.Pro364Leu)	NM_000463.3	rs34946978	4.60	2.08
*UGT1A1*	c.1456T>G (p.Tyr486Asp)	NM_000463.3	rs34993780	1.21	0.83
*SLC25A13*	c.852_855del (p.Met285ProfsTer2)	NM_014251.3	rs80338720	0.12	0
*SLC10A1*	c.800C>T (p.Ser267Phe)	NM_003049.4	rs2296651	1.94	2.50
*ABCC2*	c.2302C>T (p.Arg768Trp)	NM_000392.5	rs56199535	0	0
*G6PD*	c.871G>A (p.Val291Met)	NM_001360016.2	rs137852327	0	0
*G6PD*	c.95A>G (p.His32Arg)	NM_001360016.2	rs137852340	0	0
*G6PD*	c.1024C>T (p.Leu342Phe)	NM_001360016.2	rs137852342	0	0
*G6PD*	c.1376G>T (p.Arg459Leu)	NM_001360016.2	rs72554665	0.17	0
*G6PD*	c.1388G>A (p.Arg463His)	NM_001360016.2	rs72554664	1.05	0

#### 2.2.2 Sample collection and DNA extraction

Peripheral blood (1 mL) and two dried blood spots (8 mm) were collected from each neonate. DNA was extracted using an NP968-C automated nucleic acid extraction system and its compatible reagent kit (Xi’an Tianlong Science and Technology Co., Ltd., China) with the whole-blood extraction program. The extracted DNA was quantified using a Qubit 4.0 fluorometer, and DNA concentrations were confirmed to be within 1–100 ng/*μ*L. Samples were stored at −20 °C until analysis.

#### 2.2.3 Genetic testing

Multicolor melting curve analysis (MMCA) was used for simultaneous high-throughput genotyping of the 12 loci. Specific PCR primers and fluorescent probes were designed for each target locus and synthesized by Beijing Boao Shenghe Medical Laboratory Co., Ltd.

The PCR reaction volume was 20 μL and included template DNA, PCR premix, primers, and probes. PCR amplification and melting curve analysis were performed using an SLAN-96P automated medical PCR analysis system (Shanghai Hongshi Medical Technology Co., Ltd.; software version 8.2).

Genotypes were determined by comparing the melting curve peaks of each sample with the Tm values of known genotype controls (wild type, heterozygous, and homozygous). Approximately 10% of samples were randomly selected without stratification by genotype frequency for Sanger sequencing validation, and the concordance rate with MMCA genotyping results was 100%. All enrolled neonates had successful genotype calls for the 12 targeted loci, with no assay failures observed. In this study, detection of a targeted variant indicates the presence of the specified genetic variant and does not imply classification as pathogenic or likely pathogenic.

### 2.3 Statistical analysis

Statistical analyses were performed using SPSS version 26.0. Categorical variables are presented as counts and percentages [n (%)]. Variant allele frequencies were calculated separately for the case and control groups. For autosomal loci, the variant allele frequency was calculated as (number of heterozygotes + 2 × number of homozygotes)/(2 × total number of individuals). For the X-linked *G6PD* loci, the total number of X chromosomes (number of males + 2 × number of females) was used as the denominator. Genotype distributions were summarized as *n* (%). Carrier and genotype-specific comparisons were performed using the wild-type genotype as the reference, as applicable. Hardy-Weinberg equilibrium was assessed in controls for polymorphic autosomal loci using an exact test (Supplementary Table S2); monomorphic loci were not assessed. Between-group comparisons were performed using the chi-square test or Fisher's exact test, as appropriate. Odds ratios (ORs) and 95% confidence intervals (CIs) were calculated for associations between the investigated genetic variants and unexplained NHB. Multiple testing was controlled using the Benjamini–Hochberg false discovery rate (FDR) procedure within each analysis level, with FDR-adjusted *P* < 0.05 considered significant (Supplementary Table S3). Sensitivity analyses were restricted to the six centers that contributed both cases and controls and were performed using multivariable logistic regression models adjusted for sex and recruitment center. Recruitment center was included as a categorical variable. Exploratory follow-up data were summarized descriptively, and no inferential comparisons were performed. All tests were two-sided.

## 3 Results

### 3.1 General characteristics and overall target variant detection

A total of 413 neonates with unexplained NHB and 120 control neonates were included. Cases were recruited from seven participating centers and controls from six centers (Supplementary Table S1). Among centers contributing both groups, the distribution of cases and controls did not differ significantly (*χ^2^* = 8.85, *P* = 0.115). The case group included 253 males (61.3%) and 160 females (38.7%), whereas the control group included 72 males (60.0%) and 48 females (40.0%). All neonates completed genotyping of the 12 selected loci. The target variant detection rate was significantly higher in the case group than in the control group (63.0% [260/413] vs. 45.0% [54/120]; *OR* = 2.08, 95% *CI*: 1.38–3.13, *P* < 0.001). Among neonates with detected variants, the proportions of single-site homozygous/hemizygous variants and multi-site variants were relatively higher in the case group; however, the overall variant-type composition did not differ significantly between the groups (*χ^2^* = 4.55, *P* = 0.10). Details are shown in Table 2.

**Table 2:** Demographic and genetic characteristics of the study population.

Item	Case group (*n* = 413)	Control group (*n* = 120)	Statistic	*P* value
Sex [n(%)]			*χ^2^* = 0.06	0.80
Male	253 (61.3)	72 (60.0)		
Female	160 (38.7)	48 (40.0)		
Overall target variant detection [n(%)]			*χ^2^* = 12.38	<0.001
No target variant detected	153 (37.0)	66 (55.0)		
Target variant detected	260 (63.0)	54 (45.0)		
Variant-type composition [n(%)]			*χ^2^* = 4.55	0.10
Single-site heterozygous variant	169 (65.0)	43 (79.6)		
Single-site homozygous/hemizygous variant^a^	57 (21.9)	6 (11.1)		
Multi-site variant	34 (13.1)	5 (9.3)		

a Single-site homozygous/hemizygous variants included autosomal homozygous variants and *G6PD* c.1388G>A male hemizygous variants.

### 3.2 Gene-level distribution and association analysis of selected variants

At the gene level, *UGT1A1* variant detection rate was significantly higher in the case group than in the control group (60.3% [249/413] vs. 40.8% [49/120]; *OR* = 2.20, 95% *CI*: 1.45–3.33, *P* < 0.001). *SLC10A1* variants were detected in 3.9% of cases and 5.0% of controls. *G6PD* and *SLC25A13* variants were detected only in the case group, whereas no *ABCC2* variants were detected in either group. No significant between-group differences were observed for *SLC10A1*, *G6PD*, or *SLC25A13* variants. Details are shown in Table 3. After FDR correction for gene-level comparisons, the association of *UGT1A1* with unexplained NHB remained statistically significant (FDR-adjusted *P* = 0.00063), whereas the other gene-level comparisons remained non-significant (Supplementary Table S3).

**Table 3:** Comparison of gene-level variants.

Gene	Group	Variant-positive neonates^a^	Proportion among variant-positive neonates(%)	Within-group detection rate(%)	*OR* (95% *CI*)	*P* value
*UGT1A1*	Case group	249	95.8	60.3	2.20 (1.45–3.33)	<0.001
	Control group	49	90.7	40.8		
*SLC10A1*	Case group	16	6.2	3.9	0.77 (0.29–2.00)	0.59
	Control group	6	11.1	5.0		
*G6PD*	Case group	7	2.7	1.7	-	0.36*
	Control group	0	0	0		
*SLC25A13*	Case group	1	0.4	0.2	-	1.00*
	Control group	0	0	0		
*ABCC2*	Case group	0	0	0	-	-
	Control group	0	0	0		

FDR-adjusted *P* values are provided in Supplementary Table S3.

a The same neonate could be counted under more than one gene if variants in multiple genes were detected.

* Fisher's exact test.

### 3.3 Locus-level distribution and association analysis of selected variants

Variant allele frequencies for all 12 investigated loci in the case and control groups are presented in Table 1. Among the assessable polymorphic autosomal loci, no significant deviation from Hardy-Weinberg equilibrium was observed in the control group (all exact *P* > 0.05; Supplementary Table S2). *UGT1A1* c.211G>A was the most frequently detected locus in both groups. Carriers of at least one c.211G>A variant allele (HET + HOM) accounted for 53.3% (220/413) of cases and 37.5% (45/120) of controls (*OR* = 1.90, 95% *CI*: 1.25–2.89, *P* = 0.002). Using the wild-type genotype as the reference, the heterozygous genotype was observed in 40.7% of cases and 32.5% of controls (*OR* = 1.67, 95% *CI*: 1.08–2.60, *P* = 0.021), whereas the homozygous genotype was observed in 12.6% and 5.0%, respectively (*OR* = 3.37, 95% *CI*: 1.39–8.17, *P* = 0.005). After FDR correction, the c.211G>A carrier association remained significant, as did the heterozygous and homozygous genotype-specific comparisons (FDR-adjusted *P* = 0.0189, 0.0413, and 0.0193, respectively); no other locus-level or genotype-specific associations remained significant (Supplementary Table S3). For the primary genetic associations, sensitivity analyses were restricted to the six centers contributing both cases and controls and were performed using multivariable logistic regression models adjusted for sex and recruitment center. These analyses yielded similar effect estimates for the main associations, including overall target variant detection, *UGT1A1* variants, and *UGT1A1* c.211G>A carrier status (Supplementary Table S4).

The carrier frequency of *UGT1A1* c.1091C>T was 8.2% in cases and 4.2% in controls. Four homozygous carriers at this locus were observed only in the case group, whereas heterozygous carriers were observed in both groups; however, neither the carrier nor genotype-specific comparisons reached statistical significance (*P* > 0.05). *UGT1A1* c.1456T>G, c.686C>A, and *SLC10A1* c.800C>T were detected only as heterozygous variants, with no significant between-group differences. Six male hemizygous carriers at *G6PD* c.1388G>A, one female heterozygous carrier at *G6PD* c.1376G>T, and one heterozygous carrier at *SLC25A13* c.852_855del were observed only in the case group, but the differences were not statistically significant (*P* > 0.05). No variant alleles were detected at the remaining four loci (*G6PD* c.95A>G, c.871G>A, c.1024C>T, and *ABCC2* c.2302C>T). Details are shown in Table 4.

**Table 4:** Genotype distributions and locus-level association analyses.

Variant locus	Genotype	Case group (*n* = 413)	Control group (*n* = 120)	*OR* (95% *CI*)	*P* value
*UGT1A1* c.211G>A	Wild type	193 (46.7)	75 (62.5)	Reference	
	Heterozygous	168 (40.7)	39 (32.5)	1.67 (1.08–2.60)	0.021
	Homozygous	52 (12.6)	6 (5.0)	3.37 (1.39–8.17)	0.005
	HET + HOM	220 (53.3)	45 (37.5)	1.90 (1.25–2.89)	0.002
*UGT1A1* c.1091C>T	Wild type	379 (91.8)	115 (95.8)	Reference	
	Heterozygous	30 (7.3)	5 (4.2)	1.82 (0.69–4.80)	0.22
	Homozygous	4 (1.0)	0 (0)	-	0.58*
	HET + HOM	34 (8.2)	5 (4.2)	2.06 (0.79–5.38)	0.13
*UGT1A1* c.1456T>G	Wild type	403 (97.6)	118 (98.3)	Reference	
	Heterozygous	10 (2.4)	2 (1.7)	1.46 (0.32–6.77)	1.00*
*UGT1A1* c.686C>A	Wild type	406 (98.3)	119 (99.2)	Reference	
	Heterozygous	7 (1.7)	1 (0.8)	2.05 (0.25–16.84)	0.69*
*SLC10A1* c.800C>T	Wild type	397 (96.1)	114 (95.0)	Reference	
	Heterozygous	16 (3.9)	6 (5.0)	0.77 (0.29–2.00)	0.59
*G6PD* c.1388G>A	Male wild type	247 (97.6)	72 (100.0)	Reference	
	Male hemizygous	6 (2.4)	0 (0)	-	0.35*
*G6PD* c.1376G>T	Female wild type	159 (99.4)	48 (100.0)	Reference	
	Female heterozygous	1 (0.6)	0 (0)	-	1.00*
*SLC25A*13 c.852_855del	Wild type	412 (99.8)	120 (100.0)	Reference	
	Heterozygous	1 (0.2)	0 (0)	-	1.00*

HET, heterozygous; HOM, homozygous. HET + HOM represents carriers of at least one variant allele. For G6PD variants, genotype frequencies were calculated within the corresponding sex.

FDR-adjusted *P* values are provided in Supplementary Table S3.

* Fisher's exact test.

### 3.4 Analysis of multi-site variants

The multi-site variant detection rate was 8.2% (34/413) in the case group and 4.2% (5/120) in the control group, with no statistically significant difference (*χ^2^* = 2.27, *P* = 0.13). Further classification according to the genes involved showed that 25 neonates had multi-site variants within the same gene, including 21 cases (61.8%, 21/34) and 4 controls (80.0%, 4/5), all involving *UGT1A1*. Cross-gene multi-site variants were detected in 14 neonates, including 13 cases (38.2%, 13/34) and 1 control (20.0%, 1/5).

Among neonates with multi-site variants, combinations frequently involved *UGT1A1* c.211G>A. In the case group, the most common combination was *UGT1A1* c.211G>A + c.1091C>T, detected in 14 neonates (41.2%), followed by *UGT1A1* c.211G>A + *SLC10A1* c.800C>T in 7 neonates (20.6%) and *UGT1A1* c.211G>A + c.1456T>G in 6 neonates (17.6%). In addition, *G6PD* c.1388G>A male hemizygosity combined with *UGT1A1* c.211G>A was detected only in the case group, in 3 neonates. In the control group, multi-site variants were uncommon (*n* = 5), with *UGT1A1* c.211G>A + c.1456T>G detected in 2 neonates and each of the remaining combinations detected in 1 neonate. Details are shown in Table 5.

**Table 5:** Distribution of multi-site variant combinations.

Variant combination	Genotype pattern	Case group (*n* = 34)	Control group (*n* = 5)
Variants within the same gene		21 (61.8)	4 (80.0)
*UGT1A1* c.211G>A + c.1091C>T	Heterozygous + heterozygous	14 (41.2)	1 (20.0)
*UGT1A1* c.211G>A + c.1456T>G	Heterozygous + heterozygous	6 (17.6)	2 (40.0)
*UGT1A1* c.211G>A + c.686C>A	Heterozygous + heterozygous	1 (2.9)	1 (20.0)
Cross-gene multi-site variants		13 (38.2)	1 (20.0)
*UGT1A1* c.211G>A + *SLC10A1* c.800C>T	Heterozygous + heterozygous	7 (20.6)	1 (20.0)
*UGT1A1* c.211G>A + *SLC10A1* c.800C>T	Homozygous + heterozygous	2 (5.9)	0 (0)
*G6PD* c.1388G>A + *UGT1A1* c.211G>A	Male hemizygous + heterozygous	3 (8.8)	0 (0)
*UGT1A1* c.211G>A + c.1091C>T + *SLC10A1* c.800C>T	Triple heterozygous	1 (2.9)	0 (0)
Total		34 (100.0)	5 (100.0)

### 3.5 Exploratory follow-up of control neonates with detected target variants

Exploratory follow-up was available only for the 54 control neonates with detected target variants and was analyzed descriptively. Among these neonates, 32 (59.3%) subsequently developed NHB. Among controls with single-locus variants, NHB occurred in 21 of 39 neonates with heterozygous *UGT1A1* c.211G>A and in 4 of 6 with homozygous *UGT1A1* c.211G>A. For other single-locus variants, subsequent NHB was observed in 2 of 5 neonates with heterozygous *UGT1A1* c.1091C>T, 1 of 2 with heterozygous *UGT1A1* c.1456T>G, 0 of 1 with heterozygous *UGT1A1* c.686C>A, and 2 of 6 with heterozygous *SLC10A1* c.800C>T. Among the five control neonates with multi-site variants, three subsequently developed NHB.

## 4 Discussion

Increasing evidence supports an important role for genetic factors in NHB. In this multicenter study, bilirubin metabolism-related variants showed distinct distribution patterns among northern Chinese neonates with unexplained NHB, with *UGT1A1* variants being the most frequently detected and multi-site variant patterns observed in a subset of neonates.

*UGT1A1* variants can reduce or abolish UGT1A1 enzyme activity and constitute an important molecular basis for the phenotypic spectrum of bilirubin metabolism disorders ranging from benign Gilbert syndrome to severe Crigler-Najjar syndrome (5, 10, 11). In the present study, *UGT1A1* variants were frequently detected among neonates with unexplained NHB in northern China. *UGT1A1* c.211G>A, also known as UGT1A1*6, was the predominant variant in this study. Carriers of at least one c.211G>A variant allele were more frequent among cases than controls. In genotype-specific analyses, both heterozygous and homozygous c.211G>A genotypes were associated with higher odds of unexplained NHB and remained significant after FDR correction, with a higher *OR* observed for the homozygous genotype. This pattern may suggest a dose-related effect at this locus. Previous cross-regional studies have also suggested that *UGT1A1* c.211G>A is an important genetic risk locus for neonatal hyperbilirubinemia. Studies from Thailand and Vietnam reported a high frequency of *UGT1A1* c.211G>A among neonates with NHB and found that both homozygous and heterozygous variants were significantly associated with NHB, with total bilirubin levels being higher in homozygous than in heterozygous carriers (19, 20). A genome-wide association study by Chen et al. based on the Qingdao Birth Cohort showed that rs4148323 (i.e., c.211G>A) was one of the genetic loci most strongly associated with neonatal jaundice (*P* = 2.3 × 10⁻^18^). Notably, analysis of population data from 31 provincial-level administrative regions in China showed that the allele frequency of *UGT1A1* c.211G>A was highest in Tibet and almost undetectable in Guangxi, suggesting that population genetic background may influence the distribution of this locus (21). Together with the present findings, these data support *UGT1A1* c.211G>A as an important NHB-associated locus in East Asian populations.

In addition to c.211G>A, several other *UGT1A1* loci were examined in this study. In the case group, the carrier frequencies were 8.2% for c.1091C>T (allele frequency 4.6%), 2.4% for c.1456T > G (allele frequency 1.2%), and 1.7% for c.686C>A (allele frequency 0.8%). These loci had relatively low carrier frequencies and were not significantly associated with unexplained NHB in this study population. Mi et al. reported the distribution of these loci among patients with NHB in southeastern China, with frequencies of 4.22% for c.1091C>T, 2.82% for c.1456T > G, and 3.75% for c.686C>A (22). The frequency of c.1091C>T in that study was similar to our findings, whereas the other two loci differed to some extent, which may be related to geographic differences in the study populations or differences in sample size.

Beyond *UGT1A1*, this study also examined hotspot loci in four additional genes with different inheritance patterns and pathogenic mechanisms, highlighting the complexity of the genetic background of NHB. *SLC10A1* encodes NTCP, which is involved in hepatocellular uptake of bile acids and bilirubin. NTCP deficiency is an autosomal recessive disorder, and previous studies have shown that homozygous *SLC10A1* c.800C>T accounts for more than 90% of reported variants (23, 24). In the present study, heterozygous *SLC10A1* c.800C>T was detected in 16 neonates in the case group (3.9%), 10 of whom also carried *UGT1A1* variants; in the control group, 6 neonates (5.0%) carried this variant, and only 1 also carried a *UGT1A1* variant. These findings indicate that *SLC10A1* variants may coexist with *UGT1A1* variants in some neonates with unexplained NHB; however, the contribution of such variant combinations requires further investigation. *G6PD* is located on the X chromosome and follows an X-linked incompletely dominant inheritance pattern; male hemizygotes can be affected, whereas female heterozygotes may show variable manifestations. G6PD deficiency is an important cause of NHB, mainly by inducing hemolysis and increasing bilirubin production (25, 26). In this study, six male hemizygous carriers of *G6PD* c.1388G>A and one female heterozygous carrier of *G6PD* c.1376G>T were identified only in the case group and not in the control group. Notably, three male hemizygous carriers of *G6PD* c.1388G>A also carried heterozygous *UGT1A1* c.211G>A. These coexisting variants involve genes participating in different aspects of bilirubin metabolism, although the functional and clinical significance of their coexistence remains unclear.

In contrast, *SLC25A13* and *ABCC2* variants were rarely detected in this study, suggesting that they may be relatively uncommon in neonates from northern China. In addition, cases in the present study were characterized predominantly by unconjugated bilirubin elevation, whereas *SLC25A13* deficiency-associated NICCD and *ABCC2* deficiency-associated Dubin-Johnson syndrome typically present with intrahepatic cholestasis dominated by conjugated hyperbilirubinemia (27, 28).

Multi-site variants were observed in both case and control groups, although the difference was not statistically significant. The observed combinations included multiple loci within *UGT1A1* and cross-gene combinations involving *UGT1A1* with *SLC10A1* or *G6PD*. These findings document variant coexistence, but the limited number of multi-site carriers does not allow evaluation of additive effects or biological interactions. Because family-based verification or haplotype analysis was not performed, the phase and functional impact of *UGT1A1* variants could not be determined. In the study by Mi et al., all patients with NHB carried multiple *UGT1A1* variants (22), suggesting that multi-site *UGT1A1* variant combinations may be an important component of the genetic background of NHB in Chinese populations. In a study of cross-gene multi-site variants, Huang et al. reported that cumulative burden of bilirubin metabolism-related genetic variants was associated with increased risks of severe NHB and identified recurrent coexisting variant patterns involving *UGT1A1*, *G6PD*, and *SLC10A1* (29). Similar cross-gene variant patterns were observed in our study.

This study has several limitations. First, controls were enrolled on the day of birth and had not been diagnosed with NHB at the time of enrollment; however, they were not confirmed to remain unaffected throughout the neonatal period. Follow-up was limited to variant-positive controls, so subsequent outcomes were not ascertained uniformly across the control group. Accordingly, the primary analyses compare unexplained NHB cases with newborns without diagnosed NHB at enrollment, rather than with confirmed unaffected controls. Second, although cases were restricted to neonates with unexplained NHB after exclusion of major identifiable clinical causes, detailed quantitative clinical phenotype data were not systematically available across centers, limiting genotype-phenotype analyses. Sensitivity analyses adjusted for sex and recruitment center supported the robustness of the main findings, but residual confounding by unavailable clinical factors cannot be excluded. Third, the lack of family samples and haplotype analyses limited the determination of the phase of multiple *UGT1A1* variants and the assessment of potential compound heterozygous effects. Finally, despite the multicenter design, potential selection bias and population-structure effects cannot be completely excluded, and extrapolation to other populations should be cautious.

This multicenter case-control study characterized the distribution of 12 targeted bilirubin metabolism-related variants in northern Chinese neonates with unexplained NHB. *UGT1A1* variants were the most frequently detected. *UGT1A1* c.211G>A was the most frequently detected locus and remained associated with unexplained NHB after FDR correction. Within-gene and cross-gene multi-site variant combinations were also observed, illustrating diverse patterns of bilirubin metabolism-related genetic variation in this population. These findings provide regional data on bilirubin metabolism-related variants in northern Chinese neonates. Further prospective studies with complete follow-up, detailed clinical phenotypes, and family-based analyses are needed to determine the independent contribution and clinical relevance of these variants.

## Data Availability

The datasets presented in this article are not readily available due to ethical and privacy restrictions. Requests to access the datasets should be directed to the corresponding author.

## References

[1] Hansen TWR Wong RJ Stevenson DK. Molecular physiology and pathophysiology of bilirubin handling by the blood, liver, intestine, and brain in the newborn. Physiol Rev. (2020) 100:1291–346. 10.1152/physrev.00004.2019 32401177

[2] Teixeira MH Borges VMS Riesgo RDS Sleifer P. Hyperbilirubinemia impact on newborn hearing: a literature review. Rev Assoc Med Bras. (2020) 66:1002–8. 10.1590/1806-9282.66.7.1002 32844928

[3] Tsao P-C Yeh H-L Shiau Y-S Chang Y-C Chiang S-H Soong W-J. Long-term neurodevelopmental outcomes of significant neonatal jaundice in Taiwan from 2000 to 2003: a nationwide, population-based cohort study. Sci Rep. (2020) 10:11374. 10.1038/s41598-020-68186-w 32647318 PMC7347619

[4] Chiddarwar AS D’Silva SZ Colah RB Ghosh K Mukherjee MB. Genetic variations in bilirubin metabolism genes and their association with unconjugated hyperbilirubinemia in adults. Ann Hum Genet. (2017) 81:11–9. 10.1111/ahg.12179 27943244

[5] Bosma PJ Chowdhury JR Bakker C Gantla S De Boer A Oostra BA. The genetic basis of the reduced expression of bilirubin UDP-glucuronosyltransferase 1 in gilbert’s syndrome. N Engl J Med. (1995) 333:1171–5. 10.1056/NEJM199511023331802 7565971

[6] Agrawal SK Kumar P Rathi R Sharma N Das R Prasad R. UGT1A1 gene polymorphisms in north Indian neonates presenting with unconjugated hyperbilirubinemia. Pediatr Res. (2009) 65:675–80. 10.1203/PDR.0b013e31819ed5de 19430380

[7] Frank JE. Diagnosis and management of G6PD deficiency. Am Fam Physician. (2005) 72:1277–82. 16225031

[8] Riskin A Bravdo Y Habib C Maor I Mousa J Shahbarat S. The genetics of glucose-6-phosphate dehydrogenase and uridine diphosphate glucuronosyltransferase 1A1 promoter gene polymorphism in relation to quantitative biochemical G6PD activity measurement and neonatal hyperbilirubinemia. Children (Basel). (2023) 10:1172. 10.3390/children10071172 37508669 PMC10378156

[9] Hamoud AR Weaver L Stec DE Hinds TD Jr. Bilirubin in the liver-gut signaling axis. Trends Endocrinol Metab. (2018) 29:140–50. 10.1016/j.tem.2018.01.002 29409713 PMC5831340

[10] Memon N Weinberger BI Hegyi T Aleksunes LM. Inherited disorders of bilirubin clearance. Pediatr Res. (2016) 79:378–86. 10.1038/pr.2015.247 26595536 PMC4821713

[11] Wagner KH Shiels RG Lang CA Seyed Khoei N Bulmer AC. Diagnostic criteria and contributors to gilbert’s syndrome. Crit Rev Clin Lab Sci. (2018) 55:129–39. 10.1080/10408363.2018.1428526 29390925

[12] Vitek L Hinds TD Jr Stec DE Tiribelli C. The physiology of bilirubin: health and disease equilibrium. Trends Mol Med. (2023) 29:315–28. 10.1016/j.molmed.2023.01.007 36828710 PMC10023336

[13] Mason PJ Bautista JM Gilsanz F. G6PD deficiency: the genotype-phenotype association. Blood Rev. (2007) 21:267–83. 10.1016/j.blre.2007.05.002 17611006

[14] Jiang W Yu G Liu P Geng Q Chen L Lin Q. Structure and function of glucose-6-phosphate dehydrogenase-deficient variants in the Chinese population. Hum Genet. (2006) 119:463–78. 10.1007/s00439-005-0126-5 16607506

[15] Tan M Liu X Zhang Y Yin Y Chen T Li Y. Molecular epidemiological characteristics, variant spectrum and genotype-phenotype correlation of glucose-6-phosphate dehydrogenase deficiency in China: a population-based multicenter study using newborn screening. PLoS One. (2024) 19:e0310517. 10.1371/journal.pone.0310517 39436963 PMC11495603

[16] American Academy of Pediatrics Subcommittee on Hyperbilirubinemia. Management of hyperbilirubinemia in the newborn infant 35 or more weeks of gestation. Pediatrics. (2004) 114:297–316. 10.1542/peds.114.1.297 15231951

[17] Kemper AR Newman TB Slaughter JL Maisels MJ Watchko JF Downs SM. Clinical practice guideline revision: management of hyperbilirubinemia in the newborn infant 35 or more weeks of gestation. Pediatrics. (2022) 150:e2022058859. 10.1542/peds.2022-058859 35927462

[18] Editorial Board of Chinese Journal of Pediatrics, Subspecialty Group of Neonatology, Society of Pediatrics, Chinese Medical Association. Experts consensus on principles for diagnosis and treatment of neonatal jaundice. Zhonghua Er Ke Za Zhi. (2010) 48:685–6. 21092529

[19] Atasilp C Kanjanapipak J Vichayaprasertkul J Jinda P Tiyasirichokchai R Srisawasdi P. Associations between UGT1A1 and SLCO1B1 polymorphisms and susceptibility to neonatal hyperbilirubinemia in the Thai population. BMC Pediatr. (2022) 22:243. 10.1186/s12887-022-03311-4 35501760 PMC9059389

[20] Nguyen TT Zhao W Yang X Zhong DN. The relationship between hyperbilirubinemia and the promoter region and first exon of UGT1A1 gene polymorphisms in Vietnamese newborns. Pediatr Res. (2020) 88:940–4. 10.1038/s41390-020-0825-6 32126570

[21] Chen X Du P Li S Wang X Xu M Chu Z. Genetic and clinical determinants of neonatal jaundice and growth patterns in the qingdao birth cohort: a genome-wide association study. PLoS One. (2026) 21:e0338567. 10.1371/journal.pone.0338567 41544045 PMC12810793

[22] Mi XX Yan J Ma XJ Zhu GL Gao YD Yang WJ. Analysis of the UGT1A1 genotype in hyperbilirubinemia patients: differences in allele frequency and distribution. Biomed Res Int. (2019) 2019:6272174–9. 10.1155/2019/6272174 31467903 PMC6699345

[23] Deng L-J Ouyang W-X Liu R Deng M Qiu J-W Yaqub M-R. Clinical characterization of NTCP deficiency in paediatric patients: a case-control study based on SLC10A1 genotyping analysis. Liver Int. (2021) 41:2720–8. 10.1111/liv.15031 34369070 PMC9291912

[24] Dawson PA Lan T Rao A. Bile acid transporters. J Lipid Res. (2009) 50:2340–57. 10.1194/jlr.R900012-JLR200 19498215 PMC2781307

[25] Riskin A Gery N Kugelman A Hemo M Spevak I Bader D. Glucose-6-phosphate dehydrogenase deficiency and borderline deficiency: association with neonatal hyperbilirubinemia. J Pediatr. (2012) 161:191–196.e1. 10.1016/j.jpeds.2012.02.018 22459229

[26] Cappellini MD Fiorelli G. Glucose-6-phosphate dehydrogenase deficiency. Lancet. (2008) 371:64–74. 10.1016/S0140-6736(08)60073-2 18177777

[27] Saheki T Kobayashi K. Mitochondrial aspartate glutamate carrier deficiency as the cause of adult-onset type II citrullinemia and idiopathic neonatal hepatitis. J Hum Genet. (2002) 47:333–41. 10.1007/s100380200046 12111366

[28] Morais MB Machado MV. Benign inheritable disorders of bilirubin metabolism manifested by conjugated hyperbilirubinemia: a narrative review. United European Gastroenterol J. (2022) 10:745–53. 10.1002/ueg2.12279 35860851 PMC9486497

[29] Huang D Gu X Li W Mi H Zeng H Zhuang G. Genomic sequencing as a key primary recommendation for neonatal hyperbilirubinemia: a population-based multicenter study. J Genet Genomics. (2026) 53:467–75. 10.1016/j.jgg.2025.09.002 40939888

