# Supplementary material for: Bilirubin metabolism-related genetic variants in neonatal hyperbilirubinemia: a multicenter case-control study in northern China
Source: Front Pediatr. 2026 Oct 20;14:1945311. doi: 10.3389/fped.2026.1945311 (PMC13727740; doi:10.3389/fped.2026.1945311)
Supplement: Supplementary file 1 [file Supplementaryfile1.docx]

**Supplementary Table S1. Distribution of cases and controls across participating centers**

| **Participating center** | **Cases, n (%)** | **Controls, n (%)** | **Total, n (%)** |
| --- | --- | --- | --- |
| Center 1 | 80 (19.4%) | 0 (0.0%) | 80 |
| Center 2 | 78 (18.9%) | 17 (14.2%) | 95 |
| Center 3 | 67 (16.2%) | 29 (24.2%) | 96 |
| Center 4 | 55 (13.3%) | 26 (21.7%) | 81 |
| Center 5 | 55 (13.3%) | 15 (12.5%) | 70 |
| Center 6 | 48 (11.6%) | 16 (13.3%) | 64 |
| Center 7 | 30 (7.3%) | 17 (14.2%) | 47 |

**Supplementary Table S2. Hardy-Weinberg equilibrium assessment of polymorphic autosomal loci in the control group**

| Variant locus | Wild type, n | Heterozygous, n | Homozygous, n | Variant allele frequency (%) | HWE exact *P* value |
| --- | --- | --- | --- | --- | --- |
| *UGT1A1* c.211G>A | 75 | 39 | 6 | 21.25 | 0.785 |
| *UGT1A1* c.1091C>T | 115 | 5 | 0 | 2.08 | 1.000 |
| *UGT1A1* c.1456T>G | 118 | 2 | 0 | 0.83 | 1.000 |
| *UGT1A1* c.686C>A | 119 | 1 | 0 | 0.42 | 1.000 |
| *SLC10A1* c.800C>T | 114 | 6 | 0 | 2.50 | 1.000 |

**Supplementary Table S3. False discovery rate correction for genetic association analyses**

| **Analysis** | **Nominal *P* value** | **FDR-adjusted *P* value** |
| --- | --- | --- |
| **A. Gene-level associations** |  |  |
| *UGT1A1* | 0.000158 | 0.000630 |
| *SLC10A1* | 0.585223 | 0.780298 |
| *G6PD* | 0.358454 | 0.716909 |
| *SLC25A13* | 1.000000 | 1.000000 |
| **B. Locus-level carrier associations** |  |  |
| *UGT1A1* c.211G>A, HET+HOM vs WT | 0.002357 | 0.018855 |
| *UGT1A1* c.1091C>T, HET+HOM vs WT | 0.132199 | 0.528795 |
| *UGT1A1* c.1456T>G | 1.000000 | 1.000000 |
| *UGT1A1* c.686C>A | 0.690367 | 1.000000 |
| *SLC10A1* c.800C>T | 0.585223 | 1.000000 |
| *G6PD* c.1388G>A, male hemizygous | 0.344767 | 0.919379 |
| *G6PD* c.1376G>T, female heterozygous | 1.000000 | 1.000000 |
| *SLC25A13* c.852_855del | 1.000000 | 1.000000 |
| **C. Genotype-specific associations** |  |  |
| *UGT1A1* c.211G>A, HET vs WT | 0.020671 | 0.041341 |
| *UGT1A1* c.211G>A, HOM vs WT | 0.004820 | 0.019279 |
| *UGT1A1* c.1091C>T, HET vs WT | 0.219542 | 0.292723 |
| *UGT1A1* c.1091C>T, HOM vs WT | 0.578039 | 0.578039 |

Note. Nominal *P* values were calculated using the chi-square test or Fisher’s exact test, as appropriate. Multiple testing was addressed using the Benjamini–Hochberg false discovery rate (FDR) procedure. FDR correction was applied separately to gene-level, locus-level carrier, and genotype-specific association analyses because these represented distinct levels of genetic comparison. Monomorphic loci for which no association test could be performed were not included in the FDR correction. HET, heterozygous; HOM, homozygous; WT, wild type.

**Supplementary Table S4. Sensitivity analyses adjusted for sex and recruitment center**

| Genetic comparison | Adjusted *OR* | 95% *CI* | *P* value |
| --- | --- | --- | --- |
| Overall target variant detection | 1.81 | 1.18-2.78 | 0.007 |
| *UGT1A1* variant | 1.93 | 1.26-2.97 | 0.003 |
| *UGT1A1* c.211G>A carrier | 1.67 | 1.08-2.58 | 0.021 |

Note. Analyses were restricted to the six centers that contributed both cases and controls.
